# Erwachsenwerden mit Essstörung – Stand der Forschung und Empfehlungen für eine gelingende Transition

**DOI:** 10.1007/s00115-026-01965-4

**Published:** 2026-05-06

**Authors:** Gertraud Gradl-Dietsch, Mara Schneider, Eva Maria Skoda, Martin Teufel, Jochen Seitz, Angels Mayordomo-Aranda, Elisabeth Schulla, Silke Naab, Ulrich Voderholzer

**Affiliations:** 1 Klinik für Psychiatrie, Psychosomatik und Psychotherapie des Kindes- und Jugendalters, Kliniken und Institut der Universität Duisburg-Essen, LVR-Universitätsklinik Essen, Wickenburgstr. 21, 45147 Essen, Deutschland; 2 Klinik für Psychosomatische Medizin und Psychotherapie, Kliniken und Institut der Universität Duisburg-Essen, LVR-Universitätsklinik Essen, Essen, Deutschland; 3 Schön Klinik Roseneck, Prien am Chiemsee, Deutschland

**Keywords:** Essstörungen/Transitionsmanagement, Anorexia nervosa, Bulimia nervosa, Binge-eating-Störung, Transition in die Erwachsenenversorgung, Eating disorders/transition management, Anorexia nervosa, Bulimia nervosa, Binge eating disorder, Transition to adult care

## Abstract

**Hintergrund:**

Der Übergang in das Erwachsenenalter geht mit besonderen Herausforderungen für die Gesundheitsversorgung einher. Essstörungen treten häufig in dieser Lebensphase auf und erfordern eine kontinuierliche Behandlung, um eine Remission zu erreichen sowie Rückfälle und langfristige psychosoziale und körperliche Folgen zu reduzieren.

**Ziel der Arbeit:**

Darstellung spezifischer Herausforderungen der Transition bei Essstörungen. Ableitung von Empfehlungen zur Verbesserung des Transitionsmanagements.

**Material und Methoden:**

Narrative Übersicht über den aktuellen Forschungsstand sowie Darstellung ausgewählter Modelle der Transitionsversorgung. Auf dieser Grundlage werden praxisorientierte Empfehlungen für das Transitionsmanagement formuliert und bestehende Forschungsbedarfe aufgezeigt.

**Ergebnisse:**

Bestehende Konzepte betonen die Bedeutung eines strukturierten Übergangsmanagements, einschließlich frühzeitiger Planung, Einschätzung der Transitionsbereitschaft, aktiver Einbeziehung der Patient:innen und Angehörigen, enger Zusammenarbeit zwischen den Versorgungssystemen, standardisierter Übergabeverfahren sowie Kontinuität spezialisierter essstörungsspezifischer Therapie. Psychotherapeutische Interventionen sollten zudem entwicklungsbezogene Aufgaben der Transition adressieren, insbesondere Eigenverantwortung, Selbstmanagement, Identitätsentwicklung sowie schulische, berufliche und sozialrechtliche Aspekte. Die empirische Evidenz für die Wirksamkeit solcher Modelle ist bislang begrenzt.

**Schlussfolgerung:**

Ein strukturiertes Transitionsmanagement mit klar definierten Zuständigkeiten erscheint notwendig, um Behandlungsabbrüche, Rückfälle, Chronifizierung und die erhöhte Suizidalität in dieser Altersgruppe zu reduzieren. Gleichzeitig besteht ein erheblicher Forschungsbedarf hinsichtlich der Wirksamkeit spezifischer Transitionskonzepte, einschließlich spezialisierter Transitionsangebote oder -stationen.

**Zusatzmaterial online:**

Die Online-Version dieses Beitrags (10.1007/s00115-026-01965-4) enthält eine tabellarische Übersicht über Studien, welche die Transition bei Patienten mit Essstörungen untersucht haben.

## Abstract (en)

**Background:**

The transition to adulthood poses particular challenges for healthcare systems. Eating disorders frequently occur during this developmental period and require continuity of care in order to achieve remission and to reduce the risk of relapses as well as long-term psychosocial and physical consequences.

**Aim of the study:**

Presentation of the specific challenges of transition in cases of eating disorders and derivation of recommendations for improving transition management.

**Material and methods:**

Narrative review of the current state of research and a description of selected models of transitional care. Based on this, practical recommendations for transition management and areas requiring further research are presented.

**Results:**

Existing concepts emphasize the importance of structured transition management, including early planning, assessment of transition readiness, active involvement of patients and caregivers, close collaboration between child/adolescent and adult healthcare services, standardized handover procedures and continuity of specialized eating disorder-specific treatment. In addition, psychotherapeutic interventions should address development-related tasks of the transition, such as autonomy, self-management, identity development, and educational, vocational and socio-legal issues. Empirical evidence for the effectiveness of specific transition models remains limited.

**Conclusion:**

Structured transition management with clearly defined responsibilities appears essential to reduce treatment discontinuity, relapses, chronicity and the elevated risk of suicidality in this age group. There is a substantial need for further research on the effectiveness of transition concepts, including specialized transition programs or dedicated transition units.

Essstörungen zählen zu den häufigsten psychischen Störungen, besonders im Jugend- und jungen Erwachsenenalter. Die Anorexia nervosa (AN) hat vor allem bei Jugendlichen in den letzten Jahren an Häufigkeit zugenommen, was unter anderem auf gesellschaftliche Veränderungen und den Einfluss sozialer Medien zurückgeführt wird [12]. Themen des Übergangs vom kinder- und jugendpsychiatrischen in das Erwachsenenversorgungssystem sind von besonders großer Relevanz, da die Transitionsphase mitten in den Häufigkeitsgipfel dieser Störungen fällt. In einer Metaanalyse lag der Altersmedian für den Beginn einer AN bei 17 Jahren, der einer Bulimia nervosa (BN) bei 18 Jahren und der einer Binge-eating-Störung (BES) bei 20 Jahren [27]. Gleichzeitig ist diese Phase geprägt von wichtigen Entwicklungsschritten, die aufgrund der Auswirkungen der Essstörung nicht regelhaft durchlaufen werden.

Essstörungen sind sehr häufig mit anderen komorbiden psychischen Störungen assoziiert, besonders die AN mit vielfältigen körperlichen Folgen wie Osteoporose und Wachstumsverzögerungen [11, 30]. Die AN weist die höchste Sterblichkeitsrate aller psychischen Erkrankungen auf, mit einer standardisierten Mortalitätsrate von 5,06 %, die auf Folgen des Hungerns, aber auch auf Suizide zurückzuführen ist [14].

Die Transition in die erwachsenenpsychiatrische Versorgung stellt für viele Patient:innen und deren Angehörige eine erhebliche Belastung dar [10]. Bisherige Studien zeigen, dass die Transition bei Essstörungen noch unzureichend strukturiert und erforscht ist. Viele Betroffene erleben Versorgungslücken, Therapieabbrüche oder Rückfälle im Übergang zwischen Jugend- und Erwachsenenversorgung [2, 10, 19, 25, 29]. Katamnesestudien bei Jugendlichen mit AN zeigen nur mäßige Remissionsraten von 41 % 3 Jahre nach stationärer Behandlung [13], nach 14 Jahren sind es 49 % [23], was die Notwendigkeit unterstreicht, Transitionsprozesse zu verbessern und Behandlungsansätze gezielt an die besonderen Anforderungen und Ressourcen dieser Lebensphase anzupassen.

## Herausforderungen der Transition bei Essstörungen

Der Übergang in die Erwachsenenversorgung erfolgt in der Regel im Alter zwischen 16 und 21 Jahren. Dies fällt mit der Zeit zusammen, in der die Belastung durch psychische Störungen ihren Höhepunkt erreicht [18], und mit einer Phase, in der Jugendliche auch andere wichtige Entwicklungsschritte vollziehen. Neben den typischen Herausforderungen der Transition, die für alle behandlungsbedürftigen psychischen Erkrankungen im Jugendalter bestehen, sind mehrere Aspekte in ganz besonderer Weise für den Transitionsprozess bei Essstörungen relevant. Auswirkungen der Mangelernährung auf das Gehirn und wiederholte und langandauernde Krankenhausaufenthalte führen zu sozialer Isolation sowie zu Schwierigkeiten, (intime) Beziehungen aufzubauen. Zudem können sie die Entwicklung von Fähigkeiten verhindern, die für Autonomie, Selbstständigkeit und das Treffen eigenständiger Entscheidungen im Rahmen altersentsprechender Entwicklungsschritte erforderlich sind [8]. „Emerging adulthood“ bezeichnet eine eigenständige Entwicklungsphase im Alter von 18 bis 25 Jahren, die durch vielfältige biografische Übergänge sowie Prozesse der Exploration und Identitätsfindung gekennzeichnet ist. Dabei bewegen sich junge Menschen zwischen wachsender Autonomie und weiterhin bestehender familiärer Unterstützung [3].

Mit dem Übergang verändern sich auch die rechtlichen Rahmenbedingungen, insbesondere hinsichtlich Einwilligungsfähigkeit, Schweigepflicht, Verantwortlichkeit und Beteiligung der Angehörigen. So erhalten Betreuungspersonen aufgrund von Regelungen der Vertraulichkeit möglicherweise keine Unterstützung und Informationen mehr und fühlen sich über die Behandlung ihrer Angehörigen im Unklaren gelassen [15]. Während in der Behandlung von Jugendlichen die Eltern eine zentrale Rolle einnehmen, beispielsweise auch bei der Steuerung des Essverhaltens, liegt in der Erwachsenenversorgung der Schwerpunkt auf der Übernahme von Verantwortung durch die Patient:innen selbst [31].

Wichtige Krankheitssymptome bei AN sind geringe Krankheitseinsicht oder auch ein fehlendes Krankheitsgefühl und typischerweise eine Ambivalenz gegenüber Therapienotwendigkeit. Ein weiterer Faktor ist die bei Essstörungen, insbesondere bei AN, erforderliche medizinische Überwachung und internistische Begleitbehandlung, die ebenso wie die psychiatrisch-psychotherapeutische Behandlung auch im Erwachsenenalter fortgesetzt werden muss, um somatische Folgen zu verhindern und körperliche Gefährdungen einschätzen zu können. Gerade Essstörungen erfordern eine Spezialisierung des Behandlungskontexts, was auch in der S3-Leitlinie zu Essstörungen betont wird. Sowohl im kinder- und jugendpsychiatrischen als auch im Erwachsenenbereich bestehen große Schwierigkeiten, eine spezialisierte ambulante Therapie zu erhalten.

Eltern finden es wichtig, für eine gelungene Transition nicht nur das Alter, sondern vor allem die Bereitschaft der Patientinnen und Patienten zu berücksichtigen. Reife und die Fähigkeit zur Selbstständigkeit sind aus Sicht der Eltern zentrale Faktoren, die im Behandlungssystem bislang häufig unterschätzt werden [16]. Da viele Betroffene weiterhin im Elternhaus leben und Familien stärker in deren Alltag eingebunden sind als bei gesunden Gleichaltrigen, sollten sowohl die Bedürfnisse der Patient:innen als auch die der Eltern im Transitionsprozess berücksichtigt werden. Romantische Beziehungen können gleichermaßen eine zusätzliche Belastung als auch eine wichtige Ressource darstellen. Unterstützende Partner:innen tragen zur Stabilisierung bei und fördern die Genesung [26], eine Berücksichtigung erscheint daher sinnvoll und wichtig.

## Modelle und Konzepte der Transitionsversorgung bei Essstörungen

Essstörungsspezifische Transitionskonzepte existieren bisher nur in geringem Umfang und mit unterschiedlichen Effektstärken. Behandlungsrichtlinien orientieren sich daher an allgemeinen Konzepten für die Transition psychisch kranker junger Erwachsener [7]. Zentrale Bestandteile von Transitionskonzepten sind

- frühzeitige, niedrigschwellige Zugangsangebote für junge Erwachsene zur Überbrückung der Versorgungslücke zwischen Kinder- und Jugendpsychiatrie (KJP) und Allgemeinpsychiatrie (AP),
- eine definierte Überlappungsphase beider Systeme mit gemeinsamen Einschätzungen und klaren Kooperationsvereinbarungen sowie
- der Einsatz standardisierter Instrumente zur systematischen Erfassung von Übergangsbereitschaft, Versorgungsfähigkeit und Selbstmanagementkompetenzen [7].

In bisherigen Essstörungsleitlinien wurde das Thema der Transition noch wenig behandelt. In der englischen Leitlinie des National Institute for Health and Care Excellence (NICE) werden Übergangsprotokolle sowie eine in der Regel 6‑monatige Übergangsphase empfohlen, in der ein Therapeut aus dem Erwachsenenbereich die Patientin und deren Familie kennenlernt, um in dieser Zeit die weitere Betreuung zu planen [20].

Eine in Kanada entwickelte Intervention umfasst fünf Bausteine, die bedarfsorientiert innerhalb von 3 Monaten beliebig kombiniert werden können:

- Einstündige Online-Peer-Sitzungen („youth to youth“ und „parent to parent“)
- Transitions-Meeting mit Leitfaden und Video zur Behandlung in der AP
- Anregung zur Konsultation der Hausärzt:in (online oder in Präsenz) mit Leitfaden und Broschüre für Ärzt:innen
- Digitaler „navigation guide“ zur Planung der Transition, zu Systemunterschieden und Behandlungsoptionen

Die Studie zeigte Rekrutierungs- und Adhärenzprobleme; nur die Peer-Sitzungen und das Transitions-Meeting erreichten die vordefinierte Durchführungsquote von 75 %, die übrigen Bausteine nicht. Bei sehr kleiner Fallzahl wurden keine signifikanten Veränderungen in Essstörungssymptomatik (Eating Disorder Examination Questionnaire [EDE-Q]), Übergangsbereitschaft (Transition Readiness Assessment Questionnaire [TRAQ]) oder Veränderungsmotivation (Readiness Ruler for Eating Disorders [ED-RR]) gefunden [21].

Ein weiteres Beispiel ist das britische First-Episode-Rapid-Early-Intervention-for-Eating-Disorders(FREED)-Modell für 16- bis 25-Jährige mit Krankheitsdauer < 3 Jahren; Ziel ist die Verkürzung der unbehandelten Erkrankungsdauer und damit eine frühere Therapie zur Verbesserung der Prognose. FREED führte zu einem deutlich schnelleren Behandlungsbeginn, einer signifikanten Reduktion der „duration of untreated eating disorder“ (DUED) und dazu, dass mehr Patient:innen eine Therapie begannen als in der Kontrollgruppe [9]. Charakteristisch sind ein früher, niedrigschwelliger Zugang, eine entwicklungsadäquate Kommunikation und Therapie, der Einsatz moderner Technologien sowie schnelle, intensive Interventionen unter Einbezug des sozialen Umfelds [4].

Ein lebensspannenübergreifendes Modell spezialisierter Essstörungsversorgung („all-ages eating disorder services model“) schlägt einen Reach-down-Ansatz der AP in die KJP vor, der eine vertikale Integration über den Altersbereich von etwa 12 bis über 64 Jahren und damit eine kontinuierliche Behandlung über die gesamte Dauer der Essstörung hinweg gewährleisten würde [1]. Hier werden multiprofessionelle Teams mit Expertise in der Behandlung von Jugendlichen und Erwachsenen benötigt, sodass zur Sicherung der Versorgungskontinuität häufig zusätzliche Mittel für Personalaufbau und spezialisierte Weiterbildung in der Erwachsenenpsychiatrie erforderlich sind. Weitere Argumente für altersübergreifende Versorgungsmodelle ergeben sich daraus, dass altersgetrennte Strukturen einen frühzeitigen Behandlungsbeginn erschweren und durch getrennte Versorgungs- und Forschungsstrukturen mit kleineren Stichproben und weniger aussagekräftigen Ergebnissen die Entwicklung evidenzbasierter Konzepte behindern [28]. In einer qualitativen Studie betonten Betroffene, Bezugspersonen und Behandelnde die Bedeutung einer vorbereitenden Transitionsarbeit, die Patient:innen zur Selbststeuerung befähigt und Strategien zum Umgang mit Risiken sowie Bewältigungsstrategien vermittelt, um das Rückfallrisiko zu verringern. Gleichzeitig wurde ein Ansatz gewünscht, der komorbide Störungen und das emotionale Befinden stärker berücksichtigt. Die Jugendlichen selbst regten zudem an, Personen mit eigener Krankheitserfahrung einzubeziehen, da diese als hilfreiche Ressource erlebt werden, um praktische Orientierung zu geben und Vertrauen aufzubauen [15].

In einer weiteren Erhebung äußerten Patient:innen mit AN den Wunsch nach spezialisierten, altersgerechten Konzepten mit AN-spezifischen Einzel- und Gruppentherapien sowie praktischer Unterstützung in Alltagsfragen wie Wohnen, Arbeit/Bildung und Behördenangelegenheiten [6]. Das TRIPS-Modell (Timely Talks, Readiness, Inclusion, Preparation and Synergy; [24]) beschreibt dazu fünf evidenzbasierte Strategien zur Orientierung und Unterstützung in der Transitionsphase, die die Herausforderungen verringern und dadurch langfristig günstigere klinische Verläufe ermöglichen sollen. Konkret umfasst das Modell folgende Säulen:

- Rechtzeitige Gespräche über die bevorstehende Transition (Timely Talks)
- Einschätzung und Förderung der individuellen Übergangsreife (Readiness)
- Einbeziehung von Patient:innen und Angehörigen in den Transitionsprozess (Inclusion)
- Gezielte Vorbereitung auf die Behandlung im Erwachsenenbereich (Preparation)
- Enge Zusammenarbeit zwischen Versorgungssystemen (Synergy)

Weitere Informationen bietet Tab. 1.

**Tab. 1:** TRIPS-Modell

Prinzip	Leitgedanke	Praktische Umsetzung
*T*imely Talks	Frühzeitige Gespräche über den bevorstehenden Übergang; Beziehungsaufbau, Motivationsförderung	Regelmäßige Gespräche (mindestens 3) über Ziele, Sorgen und Erwartungen initiieren
*R*eadiness	Übergangsreife erfassen (Autonomie, Einsicht, Motivation, Lebensziele)	Nutzung standardisierter Instrumente, gezielte Förderung fehlender Kompetenzen
*I*nclusion	Betroffene, Angehörige und beide Versorgungsbereiche aktiv einbeziehen (Psychoedukation, rechtliche Rahmenbedingungen)	Angehörige als Ressource, offene Kommunikation
*P*reparation	Strukturierte Vorbereitung sichern. Rollenwechsel Angehöriger (Steuerung → Unterstützung)	Individuelle Behandlungsinformationen vermitteln; Selbstmanagementfähigkeiten fördern; Selbsthilfegruppen
*S*ynergy	Unterschiede zwischen Versorgungssystemen bewusst machen, Behandlungen möglichst gut aufeinander abstimmen	Netzwerkstrukturen, Übergangslotsen, feste Ansprechpartner

## Diskussion

Die Evidenzlage zu Transitionsprozessen bzw. zur Wirksamkeit von Transitionsmodellen bei Essstörungen ist bislang begrenzt und stellt daher eine Herausforderung für die künftige Forschung dar. Auf Basis bisheriger Erfahrungen mit Transitionsmodellen können die in Tab. 2 zusammengefassten Empfehlungen gegeben werden.

**Tab. 2:** Empfehlungen für den Transitionsprozess bei Essstörungen

*Strukturell*
Spezialisierte essstörungsspezifische Angebote in KJP und AP
Adoleszenten- bzw. Transitionsstationen und spezialisierte Ambulanzen
Verbindliche Übergabestrukturen (gemeinsame Fallgespräche, Übergabedokumente, Konferenzen)
Einsatz von Transitionskoordinator:innen
Einbindung der Kinder‑/Hausärzt:innen
*Patient:innenbezogen*
Erfassung der Transitionsbereitschaft (z. B. TRAQ [32])
Flexible Gestaltung des Übergangszeitpunkts
Förderung von Eigenverantwortung und Selbstmanagement, partizipative Entscheidungsfindung
Bearbeitung zentraler Entwicklungsaufgaben in „emerging adulthood“ (Individuation, Entwicklung sozialer Fertigkeiten, Verantwortungsübernahme, Eigenständigkeit, Identitätsentwicklung, sozialrechtliche Anforderungen der Volljährigkeit)
Einbezug signifikanter Bezugspersonen nach individuellem Entwicklungsstand/Lebenslage
Angepasste psychosoziale Begleitung (z. B. Wohnen, Ausbildung, Behandlung)

*AP* Allgemeinpsychiatrie, *KJP* Kinder- und Jugendpsychiatrie, *TRAQ* Transition Readiness Assessment Questionnaire

Insbesondere ist die Notwendigkeit von Unterstützung prozesshaft und nicht statisch zu sehen. So können zu unterschiedlichen Zeitpunkten der Transition völlig unterschiedliche Bedarfe und Bedürfnisse vorhanden sein. In künftigen Studien sollte untersucht werden, ob ein strukturiertes Transitionsmanagement und/oder spezifische Transitionsstationen klinische Outcomes und die Behandlungskontinuität im Vergleich zur Regelversorgung verbessern und welche Faktoren dabei entscheidend sind. Auch fehlt die Entwicklung und Evaluation manualisierter Programme, die entwicklungsrelevante Aufgaben des jungen Erwachsenenalters wie Werteklärung, Identität, Autonomie und Zukunftsplanung berücksichtigen [22]. Peer-basierte Modelle, bei denen junge Menschen mit eigener Behandlungserfahrung durch das Teilen der eigenen Erfahrung Hoffnung, Zugehörigkeit und Behandlungsmotivation fördern können [5], sollten speziell im Transitionskontext erforscht werden, ebenso die Bedeutung der Angehörigenarbeit, deren Rolle in der Transition kaum erforscht ist und zu der keine speziell zugeschnittenen evidenzbasierten Interventionen vorliegen [7]. Mit dem Wechsel in die Erwachsenenbehandlung bricht die Einbindung der Bezugspersonen oft abrupt ab, während Eltern Informationsdefizite und Überforderung berichten [17].

## Fazit für die Praxis

- Essstörungen sind schwere psychische Erkrankungen, die gerade in der Transitionsphase besonders häufig sind.
- Ein strukturiertes Transitionsmanagement mit eindeutig definierten Zuständigkeiten ist sinnvoll, um Rückfälle und Chronifizierung zu reduzieren, die Entwicklungschancen der Betroffenen zu verbessern und nicht zuletzt auch die bei Essstörungen relevante Suizidgefährdung zu mindern.
- Ein gelingender Übergang ist nicht nur eine organisatorische, sondern vor allem eine therapeutische Aufgabe, die Kontinuität, Beziehung und Stabilität erfordert.

## Supplementary Information

Tabelle e1: Studien, welche die Transition bei Patienten mit Essstörungen untersucht haben.

## Data Availability

Für diesen Beitrag wurden von den Autorinnen und Autoren keine neuen Daten erhoben oder generiert. Die zugrunde liegenden Informationen stammen aus bereits veröffentlichten Studien, die in diesem Artikel zitiert sind.
