# Supplementary material for: Transition to adulthood with an eating disorder—Current state of research and recommendations for successful transition
Source: Nervenarzt. 2026 May 6;97(3):252–7. [Article in German] doi: 10.1007/s00115-026-01965-4 (PMC13171660; doi:10.1007/s00115-026-01965-4)
Supplement: Supplementary file 1 — Tabelle e1: Studien, welche die Transition bei Patienten mit Essstörungen untersucht haben. [file 115_2026_1965_MOESM1_ESM.pdf]

Ergänzendes Material

Tabelle e1: Studien, die die Transition bei Patienten mit Essstörungen untersucht haben

Autor	Jahr	Inhalt	Stichprobe	Alter in Jahren	Studiendesign (Fragebogen, Interview, etc.)	Ergebnis
Arcelus et al.	2008	Aufgrund uneinheitlicher Überweisungs-prozesse und starrer Alters-grenzen ist die Transition von jugend- zum erwachsenen-psychiatrischen Bereich bei Essstörungen häufig problematisch	*N* = 206 Patienten mit Anorexia Nervosa, Bulimia Nervosa, Binge-Eating-Störung sowie nicht näher bezeichnete Essstörungen	16 bis 25 (*M* = 19.9)	Retrospektives Review über einen Zeitraum von vier Jahren (Nutzung von Falldokumenta-tionen)	Betonung der Bedeutung gut geplanter Transitionen und besonderer Herausforderungen wie Selbstwertprobleme und Reifungs-prozesse bei jungen Patienten
Lockertsen et al.	2020	Erfahrungen von Patienten mit AN beim Übergang von der kinder- und jugend-psychiatrischen hin zur erwachsenen-psychiatrischen Versorgung	*N* = 10 Patienten mit Anorexia Nervosa	19 bis 29 (*M* = 22)	Qualitative Interviews → semistrukturierte, vertiefende Interviews; dialektische, mehrstufige Fokusgruppen	Berücksichtigung der Bedürfnisse der Patienten während der Transition sowie deren Bereitschaft für die Transition ist essenziell. Patienten und Angehörigen sollten Unterschiede in der Weiterbehandlung genau erklärt werden.
McClelland et al.	2020	Untersuchung, wie viele Jugendliche mit Essstörungen nach der kinder- und jugend-psychiatrischen Behandlung noch im jungen Erwachsenen-alter psychische Hilfe nutzen, welche Angebote sie in Anspruch nehmen und, welche Faktoren das beeinflusst	*N* = 322 Patienten mit Anorexia Nervosa, Bulimia Nervosa, nicht näher bezeichnete Essstörungen (EDNOS) sowie nicht näher bezeichnete Fütter- und Essstörungen (UFED)	13 bis 17 (Diagnose im Jugend-alter)<br><br>18 und 25 (aktuelle Nutzung von Hilfs-angeboten)	Retrospektives Review junger Erwachsener, die im Jugendalter eine Essstörungs-diagnose erhalten hatten → Vergleich mit aktueller Nutzung von Hilfsangeboten	Nur wenige Patienten aus der kinder- und jugend-psychiatrischen Behandlung erhielten im jungen Erwachsenenalter noch Hilfe. Faktoren aus der früheren Behandlung beeinflussen die spätere Nutzung.
Mooney et al.	2023	Untersuchung, inwiefern psychoedukative Angebote Jugendliche während der Transition unter-stützen könnten, und wie diese gestaltet sein sollten	*N* = 6 Patienten mit Anorexia Nervosa, atypischer Anorexia Nervosa und/oder Bulimia Nervosa	Keine Angabe	Qualitative Interviews → semistrukturiert, mit den jungen Erwachsenen (ca. 30 Minuten)	Psychoedukative Angebote wurden als hilfreich für die Transition in die Erwachsenenver-sorgung bewertet
Nadarajah et al.	2021	Erfassung hinderlicher und unterstützender Faktoren für eine erfolgreiche Transition sowie Vorschläge für	*N* = 5 Patienten mit Essstörungs-diagnose	Patienten 17 bis 18 (*M* = 17.4)	Qualitative Interviews → semistrukturierte, vertiefende Interviews mit	Eingeschränktes Verständnis bzgl. der Transition. Es handelt sich oft um eine Lücke im

		Maßnahmen, die eine erfolgreiche Transition fördern könnten	(nicht genauer definiert)<br><br>*N* = 5 Bezugsper-sonen	Bezugsper-sonen 46 bis 49 (*M* = 47.3)	Jugendlichen sowie deren Eltern (30 bis 60 Minuten)	System. Notwendig, Interventionen für eine leichtere Transition zu entwickeln
Scanferla et al.	2023	Subjektive Erfahrungen von Patientinnen mit Essstörungen und ihren Familien bei der Transition in die Erwachsenen-behandlung erfassen und verstehen, wie dieser Prozess wahrgenommen wird	*N* = 12 Patienten mit Anorexia Nervosa und/oder Bulimia Nervosa<br><br>*N* = 6 Bezugsper-sonen	Patienten 19 bis 30 (*M* = 22.5)	Qualitative Interviews → semistrukturierte, vertiefende Interviews	Transition ist meist herausfordernd. Daher wird mehr Unterstützung im Transitionsprozess benötigt und individuelle Bedürfnisse der Jugendlichen sollten stärker beachtet werden.
Wales et al.	2021	Untersuchung von Erfahrungen, Hürden und unterstützenden Faktoren der Transition bei Essstörungen in Großbritannien	*N* = 5 Patienten mit Essstörungen (nicht genauer definiert)<br><br>*N* = 22 Behandler<br><br>*N* = 6 Bezugsper-sonen	Keine Angabe (aber Patienten mussten sich bereits in der Er-wachsenen-versorgung befinden)	Qualitative Interviews → semistrukturiert (ca. 50 Minuten)<br><br>4 Fokusgruppen, die von den Behandlern gehalten wurden (ca. 60 Minuten)	Verbesserungen können durch eine gute Kommunikation, eine Klärung der Unterschiede zwischen den Versorgungssyste-men sowie durch Flexibilität beim Zeitpunkt des Übergangs erzielt werden
Wales et al.	2022	Untersuchung der Wichtigkeit verschiedener Aspekte des Transitions-prozesses bei Essstörungen zur Verbesserung der Versorgungs-kontinuität	*N* = 12 Patienten mit Essstörungen (nicht genauer definiert)<br><br>*N* = 8 Behandler<br><br>*N* = 8 Bezugsper-sonen/Eltern	Keine Angabe (Patienten mussten bereits volljährig sein)	Q-Sort-Methode → Beurteilung von 40 Aussagen zum Thema Transition	Frühzeitige, koordinierte Übergänge unter Einbeziehung aller Beteiligten und eine gute Kommunikation sind entscheidend für einen erfolgreichen Übergang in die Erwachsenenversor-gung
Gilsbach et al.	2024	Übergang von stationär behandelten AN-Patientinnen aus der KJP in die Erwachsenenpsychiatrie	N = 32 weibliche ehemalige stationäre Patientinnen mit AN	18 bis 22 (M = 20.3 Jahre)	Mixed-Methods: semistrukturierte Interviews (telefonisch oder vor Ort) + demografische und klinische Daten	Mehrheit noch in KJP, viele haben Ängste vor dem Übergang (Verlust der Therapeutin, Überforderung, Angst vor schwer Erkrankten). Wunsch nach spezialisierten Angeboten für junge Erwachsene. Übergang sollte sich am Entwicklungsstand, nicht am Alter orientieren.
